# *Plasmodium* spp. in macaques, *Macaca fascicularis,* in Malaysia, and their potential role in zoonotic malaria transmission

**DOI:** 10.1051/parasite/2022032

**Published:** 2022-06-08

**Authors:** Noorazian Md Yusuf, Jannah Zulkefli, Adela Ida Jiram, Indra Vythilingam, Shamilah Hisam, Renuka Devi, Afiqah Salehhuddin, Nurulshuhada Md Ali, Maccallyster Isa, Norwahida Alias, Nurhainis Ogu salim, Adli Abd Aziz, Lokman Hakim Sulaiman

**Affiliations:** 1 Parasitology Unit, Infectious Disease Research Centre, Institute for Medical Research, Block C3 & C7, Level 2, National Institutes of Health (NIH), Ministry of Health Malaysia No. 1 Jalan Setia Murni U13/52, Seksyen U13, Bandar Setia Alam 40170 Shah Alam Selangor Malaysia; 2 Department of Parasitology, Faculty of Medicine, University of Malaya 50603 Kuala Lumpur Malaysia; 3 School of Biology, Faculty of Applied Sciences, Universiti Teknologi MARA Cawangan Negeri Sembilan Kampus Kuala Pilah, Pekan Parit Tinggi 72000 Kuala Pilah Negeri Sembilan Malaysia; 4 Centre for Environmental and Population Health, Institute for Research, Development, and Innovation, and Department of Community Medicine, School of Medicine, International Medical University No. 126, Jalan Jalil Perkasa 19, Bukit Jalil 57000 Kuala Lumpur Malaysia

**Keywords:** Zoonotic malaria, Macaques, Simian malaria, *Plasmodium knowlesi*, *Plasmodium cynomolgi*

## Abstract

Macaques, *Macaca fascicularis*, are a known reservoir of *Plasmodium knowlesi,* the agent of simian malaria which is the predominant zoonotic species affecting humans in Malaysia and other Southeast Asian countries. Recently, a naturally acquired human infection of another simian malaria parasite, *P. cynomolgi* has been reported. Thus, it is crucial to study the distribution of simian *Plasmodium* infections with particular attention to the macaques. Four hundred and nineteen (419) long-tailed macaques (*Macaca fascicularis*) were trapped in selected areas where human cases of *P. knowlesi* and *P. cynomolgi* have been reported. Nested polymerase chain reaction (PCR) was conducted to identify the *Plasmodium* spp., and circumsporozoite protein (CSP) genes of *P. knowlesi* samples were sequenced. *Plasmodium cynomolgi* infection was shown to be the most prevalent among the macaque population (68.4%). Although 50.6% of analyzed samples contained single infections either with *P. knowlesi*, *P. cynomolgi*, *P. inui*, *P. coatneyi*, or *P. fieldi*, mixed infections with double, triple, quadruple, and all 5 species were also detected. Infection with *P. cynomolgi* and *P. knowlesi* were the highest among Malaysian macaques in areas where humans and macaques are in close contact. The risk of zoonotic infection in these areas needs to be addressed since the number of zoonotic malaria cases is on the rise. With the elimination of human malaria, the risk of humans being infected with simian malaria is very high and steps should be taken to mitigate this issue.

## Abstract (fr)

Les macaques, *Macaca fascicularis*, sont un réservoir connu de *Plasmodium knowlesi*, l’agent du paludisme simien qui est l’espèce zoonotique prédominante affectant les humains en Malaisie et dans d’autres pays d’Asie du Sud-Est. Récemment, une infection humaine acquise naturellement par un autre parasite du paludisme simien, *P. cynomolgi*, a été signalée. Ainsi, il est crucial d’étudier la distribution des infections simiennes à *Plasmodium* avec une attention particulière pour les macaques. Quatre cent dix-neuf (419) macaques à longue queue (*Macaca fascicularis*) ont été piégés dans des zones sélectionnées où des cas humains de *P. knowlesi* et *P. cynomolgi* avaient été signalés. La réaction en chaîne par polymérase (PCR) nichée a été menée pour identifier les *Plasmodium* spp. et les gènes de la protéine circumsporozoïte (CSP) des échantillons de *P. knowlesi* ont été séquencés. L’infection à *P. cynomolgi* s’est avérée la plus répandue parmi la population de macaques (68,4 %). Bien que 50,6 % des échantillons analysés montraient des infections simples avec soit *P. knowlesi*, *P. cynomolgi*, *P. inui*, *P. coatneyi* ou *P. fieldi*, des infections mixtes avec deux, trois, quatre ou même les cinq espèces ont également été détectées. L’infection par *P. cynomolgi* et *P. knowlesi* était la plus élevée parmi les macaques malais dans les zones où les humains et les macaques sont en contact étroit. Le risque d’infection zoonotique dans ces zones doit être pris en compte car le nombre de cas de paludisme zoonotique est en augmentation. Avec l’élimination du paludisme humain, le risque d’être infecté par le paludisme simien est très élevé et des mesures doivent être prises pour atténuer ce problème.

## Introduction

The World Health Organisation (WHO) estimated that there were 228 million global malaria cases reported in 2018 with around 405,000 deaths, including a significant number of children under the age of 5 in the WHO Africa Region [48]. In addition to the four common species of *Plasmodium* that cause malaria in humans, simian malaria such as *Plasmodium inui* [11], *P. cynomolgi* [10, 12, 16, 38], *P. brasilianum* [24], *P. schwetzi* [12], *P. simium* [4, 15]*,* and *P. knowlesi* [5, 9, 36] can also cause malaria ins human through mosquito bites, both naturally and experimentally.

Simian or primate malaria was first reported in Malaysia in 1908 but only gained prominence in the 1960 s after the accidental discovery that *P. cynomolgi* could be transmitted to humans via mosquito bites in the laboratory [16]. In 1967, Chin and colleagues showed that *P. knowlesi* could also be transmitted in the same way [5, 6]. Naturally acquired human cases of *P. knowlesi* in Malaysia were initially thought to be extremely rare with only two reports of such cases in Peninsular Malaysia, the first of which was reported in 1965 in Pahang [5], followed by the second case five years later in Johor [17]. Subsequently, a large-scale study was conducted in Pahang to investigate whether malaria was zoonotic. However, no evidence of zoonotic *P*. *knowlesi* transmission was found at that time [9].

Four decades later, a large cluster of human infections caused by *P. knowlesi* was reported in the Kapit Division of Sarawak, Malaysian Borneo [36]. Currently, *P. knowlesi* has been reported in all countries in Southeast Asia, except Timor Leste [45]. Although *P. knowlesi* is the most important simian malaria species due to its life-threatening infection, other simian malaria species cannot be taken lightly. *Plasmodium cynomolgi* is capable of being transmitted from simian species to humans by mosquito bite in the laboratory [16]. However, the first naturally acquired *P. cynomolgi* infection in humans in Malaysia was reported in 2014 [38]. Currently, cases of *P. cynomolgi* have also been reported from Sabah and Sarawak (Malaysian Borneo), Cambodia, and Thailand [18, 19, 21, 37]. Additionally, a Danish tourist who visited Thailand and Peninsular Malaysia was reported with *P. cynomolgi* on return to Denmark [19, 31].

Malaysia is on track for malaria elimination: cases of *P. falciparum* and *P. vivax* have decreased drastically, but *P. knowlesi* is on the increase [7].

It is vital to determine the vectors of simian malaria and studies have therefore been conducted both in Peninsular Malaysia and Malaysian Borneo [8, 23, 38, 40, 41, 44, 47]. Following this, *Anopheles latens* has been incriminated as the vector in Sarawak [44] and *An. balabacensis* in Sabah in Malaysian Borneo [47], whereas *An. cracens* and *An. introlatus* are the main vectors in Kuala Lipis, Pahang [43], and Selangor, Peninsular Malaysia, respectively [44].

Although a plethora of primates, including apes, gibbons and macaques, are known to harbor malaria parasites, zoonotic *P. knowlesi* was mainly discovered among crab-eating macaques, *Macaca fascicularis*, in Malaysia, Cambodia, Indonesia, Laos, Thailand, the Philippines, and Singapore [9, 26, 30]. In a review on simian malaria in wild macaques in Southeast Asia by Jeyaprakasam, 2020, the author described in detail the simian *Plasmodium* positivity rate and bionomics of the vectors. Studies carried out in Malaysia, Indonesia, the Philippines, Singapore, and Thailand have reported a high rate of infection among the wild macaques collected in each country (ranging from 0 to 100%) [22]. Simian malaria was found in wild macaques collected from more than one locality, whereas an infection rate of more than 50% was reported among wild macaques from only one locality in Laos and Cambodia (81.5% and 68.2%, respectively), which showed a greater risk of *P. cynomolgi* transmission to humans in the future. Other than *Macaca fascicularis,* Coatney and colleagues (1971) also described other known natural hosts for simian malaria, including pig-tailed macaque, *M. nemestrina*, and leaf macaque, *Presbytis melalophos* [9]. Lee and colleagues (2011) have examined the circumsporozoite protein (CSP) gene and mitochondrial DNA of *P. knowlesi* isolates of human and *M. fascicularis* samples in selected areas in Sarawak, Malaysia Borneo to establish their genotypes and to track the emergence of the parasite. They revealed that of 108 wild *M. fascicularis* captured, the prevalence of *Plasmodium spp*. ranged from 4% to 82%, with *P. inui* being the most commonly detected [25]. Another report by Akter also showed that out of 70 *M. fascicularis* trapped, 50% were positive with *Plasmodium spp.* [1]. This was in agreement with Lee who identified *P. inui* as the most prevalent among other species [25]. This current study aimed to describe the distribution of simian malaria in *M. fascicularis* in Peninsular Malaysia, taking into account the geographical localities where cases of zoonotic malaria infection have been reported.

## Material and methods

### Ethics approval

This study was approved by the Medical Research and Ethics Committee (MREC), Ministry of Health Malaysia (MOH) with permission to use existing archived macaque blood samples as well as to collect new blood samples (NMRR-14-213-19692, ref no. KKM/NIHSEC/P14-340). The collection of macaque blood samples was approved by the Animal Care and Use Committee (ACUC) [ACUC/KKM/02(2/2014)], while the license for macaque trapping and blood collection was obtained from the Wildlife Department [PERHILITAN: PHL&TN(IP):80-54/2 Jld 21 (8)].

### Study site

**Figure 1:**
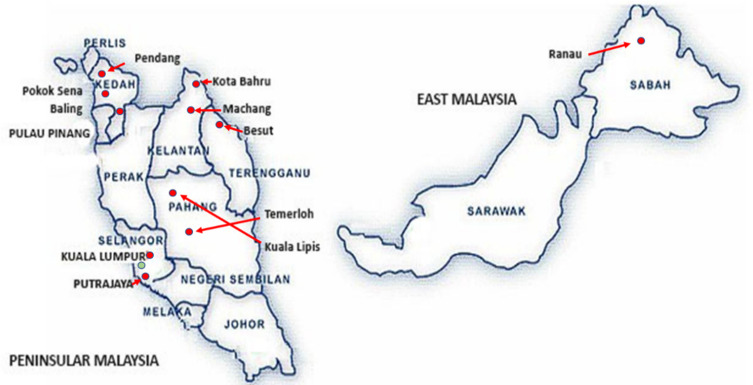
Map of Malaysia showing the study sites marked with red dots. Twelve localities were selected due to the presence of human-macaque co-habitation as well as areas with a high number of malaria cases and neighboring districts.

The *Macaca fascicularis* were trapped in selected areas in the states of Kedah, Pahang, Kelantan, Terengganu, Selangor, Kuala Lumpur, Putrajaya, and Sabah (Fig. 1) from July 2016 to January 2019. The localities were selected based on the reports of *P. knowlesi* cases in areas with proximity to human settlements. Although there were on average 2–3 cases per year in Kedah and Terengganu, and virtually no cases were reported in the Federal Territory of Kuala Lumpur and Putrajaya, the presence of a large macaque population in certain localities within the state/region was considered a risk factor for zoonotic malaria by the Wildlife Department and the Ministry of Health Malaysia. Therefore, these four states were included and noted as low endemic areas for *P. knowlesi* infection. In addition, the Wildlife Department also trapped and collected blood from macaques in Hulu Terengganu and Marang in Terengganu state. These two localities were specially selected by the Wildlife Department of Terengganu on account of the first report of *P. cynomolgi* naturally acquired human infection in 2014 [38].

### Macaque trapping

**Figure 2:**
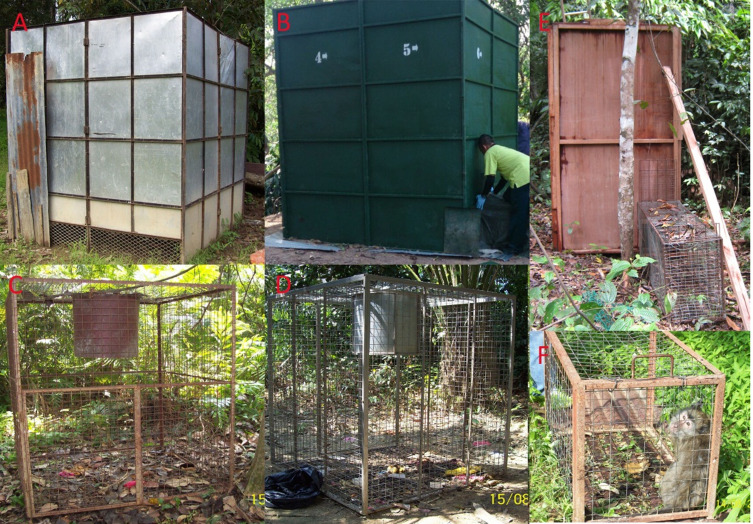
Types of monkey traps used in this study.

Several types of traps were set up in the study areas by the staff of the Wildlife Department (Fig. 2). The sizes of the traps varied from 3 × 6 × 8 feet to 8 × 8 × 9 feet. Fruits such as bananas, jackfruit, and sweet potatoes were used as bait. The trapping was done by the Wildlife Department of the respective states. Type A and B traps were made of steel sheets with no rooftop and a small door to transfer trapped macaques from the main traps to the carrier cage/transporter. The trap has an opening at the bottom covered by an iron net for inspection purposes. Type C and D traps were made from an iron net with an iron sheet made into a drum shape. Once the macaques are trapped, they cannot escape due to the smooth surface of the iron drum. The advantage of using type C and D traps was that it is easy to attract macaques due to the visibility of bait from far away. Type E was made from wooden planks with openings on the roof. The F-type served as a carrier cage for transporting trapped macaques to the blood collection center.

### Collection of blood

Trapped macaques were sedated with 0.3–0.5 mL ketamine (Vetoquinol, UK) via intramuscular injection before blood collection (1–5 mL). The blood samples were transferred into ethylenediaminetetraacetic acid (EDTA) tubes (Becton-Dickinson, Franklin Lakes, NJ, United States). Blood spots on filter paper (Whatman^®^ No. 1) were also collected. Thick and thin blood films were also prepared for malaria parasite examination by microscopy (BFMP). Each macaque was marked and then handed over to the Wildlife Department for release into the deep forest or areas of less conflict with humans. The blood samples in EDTA tubes were kept at 4 °C during transportation to the Parasitology Laboratory, IMR. Thin blood smears were fixed with methanol. Both thick and thin blood smears were then stained with 3–10% KaryoMAX^®^ Giemsa Stain (Gibco^®^, Life Technologies Carlsbad, CA, United States) for 30–60 min, examined and results were recorded. Blood samples in EDTA tubes were subjected to DNA extraction.

### DNA extraction

Two hundred microlitres of each EDTA blood sample was subjected to genomic DNA (gDNA) extraction using a DNeasy Blood and Tissue Kit (Qiagen, Hilden, Germany), following the manufacturer’s protocol. The concentration and purity of extracted DNA were determined using a NanoDrop™ 2000/200c Spectrophotometer (Thermo Fisher Scientific. Madison, WI, USA).

### Nested PCR for *Plasmodium* genus and species identification

Nested PCR was conducted to identify the genus and species of the parasites present in the blood samples pre-determined as malaria-positive by microscopy (BFMP). In all, 10% of malaria-negative samples by BFMP were selected randomly for validation by nested PCR. PCR runs were performed according to the protocol from Singh [36] to Lee [25] with slight modifications in PCR reactions. The first stage of nested PCR involved amplification of the *Plasmodium* small subunit ribosomal RNA (SSU rRNA) using the primer pair rPLU1 and rPLU5 in a 50 μL reaction volume. In the second stage, PCR was carried out for species-specific identification using primer pairs Kn1f and Kn3r for *P. knowlesi*, CY2F and CY4R for *P. cynomolgi*, PinF2 and INAR3 for *P. inui*, PfldF1 and PfldR2 for *P. fieldi*, and PctF1 and PctR1 *for P. coatneyi*, and the PCR product from the first stage as the DNA template. The master mix contained 1x reaction buffer (MyTaq™ Red Mix, 2X from Bioline, London United Kingdom), 20 nM of each primer, 200 ng DNA template, and nuclease-free water in a 50 μL reaction volume.

### Sequencing and analysis of CSP genes

The *P. knowlesi* circumsporozoite protein (CSP) genes were amplified by PCR using the primers PKCSPF2 and PKCSPR3, cloned and sequenced by the protocols described by Singh [36], Vythilingam [43], and Jiram [23]. The *CSP* gene sequences were analyzed as described previously [12, 26]. Sequences from the 456 nucleotides that encode the non-repeat N-terminal (first 195 nucleotides of the coding sequence) and C-terminal (the last 261 nucleotides of the coding sequence) regions of the *CSP* genes were aligned with Clustal W using BioEdit Sequence Alignment Editor and phylogenetically compared to those in GenBank. The phylogenetic trees were constructed using (A) the neighbor-joining (NJ) method by MEGA version 7.0 software [39] and analyzed with the Kimura-2 parameter model including transitions and transversions; (B) the Bayesian method MrBayes free software which used Markov chain Monte Carlo (MCMC) methods to estimate the posterior distribution of model parameters and, (C) Maximum Likelihood by MEGA version 7 software.

The *CSP* reference sequences were obtained from GenBank as follows; *P. knowlesi* Thailand origin (JF923566.1), *P. knowlesi* Peninsular Malaysia origin (EU687469.1, EU687467.1, EU821335.1, EU687470.1, EU EU708437.1), *P. knowlesi* Perak origin (M11031.1), *P. coatneyi* Hackeri strain Selangor, Malaysia origin (AY135360.1), *P. simium* Brazil origin (L05068.1, L05069.1), *P. cynomolgi* Berok strain Perak, Malaysia origin (M15104.1), *P. simiovale* Papua New Guinea origin (U09765.1), *P. berghei* Democratic Republic of Congo origin (X17606.1, M14135.1), *P. falciparum* Thailand origin (AB121024.1), *P. brasilianum* Venezuela strain (KM016332.1), *P. brasilianum* Brazil origin (KC906710.1), *P. malariae* Cameroon origin (AJ001523.1), *P. malariae* Uganda origin (J03992.1).

## Results

### Prevalence of simian malaria parasites in Malaysian macaques

A total of 419 *Macaca fascicularis* were trapped in selected localities in the states of Kedah, Pahang, Kelantan, Terengganu, Selangor, Kuala Lumpur, Putrajaya, and Sabah from July 2016 to January 2019 (Fig. 1). Malaria cases in Kuala Lipis had been among the highest in Peninsular Malaysia. A high number of infections by *P. knowlesi* was also reported in Kelantan state, hence the capital city, Kota Bharu, and its neighboring district Machang were selected as well as Besut in the state of Terengganu. Selangor, being the most developed state, borders Pahang and human-monkey co-habitation in certain specific localities is feared to carry a high risk of zoonotic malaria infection. The numbers and proportions of captured macaques from each state and their *Plasmodium spp.* detection are shown in Table 1.

**Table 1:** *Plasmodium* spp. detected by microscopy and PCR. Pahang, Kelantan and Terengganu have been classified as high endemic areas for *P. knowlesi* infection. Meanwhile, Kedah, Selangor, Putrajaya, and Kuala Lumpur have been described as low-endemic areas despite co-existing macaques and the human population.

Locality	Sample collected, *N* (%)	BFMP		PCR for *Plasmodium* spp.	
		+ve (%)	−ve (%)	+ve (%)	−ve (%)
Kedah	59 (14.1)	0 (0.0)	59 (100)	0 (0.0)	59 (100)
Kelantan	22 (5.1)	2 (9.1)	20 (90.9)	2 (9.1)	20 (90.9)
Terengganu	58 (13.8)	19 (32.8)	39 (67.2)	18 (31.0)	40 (69.0)
Pahang	188 (44.9)	184 (97.9)	4 (2.1)	176 (93.6)	12 (6.4)
Selangor	56 (13.4)	0 (0.0)	56 (100)	0 (0.0)	56 (100)
Sabah	4 (1.0)	4 (100)	0 (0.0)	4 (100)	0 (0.0)
W.P. Putrajaya	2 (0.5)	0 (0.0)	2 (100)	0 (0.0)	2 (100)
W.P. Kuala Lumpur	30 (7.2)	2 (6.7)	28 (93.3)	2 (6.7)	28 (93.3)
Total	419 (100.0)	211 (50.4)	208 (49.7)	202 (48.2)	217 (51.8)

211 of 419 (50.4%) samples collected were positive by BFMP, of which only 202 samples revealed positive amplification for the *Plasmodium* genus by nested PCR. Nine of the samples encountered problems during PCR due to the low concentration of DNA and contaminants. The largest number of samples were collected from Pahang and more than 90% tested positive by both methods. This is followed by Terengganu (30%) and Kelantan (9%). Although there were only 4 samples collected from Sabah, all tested positive by both methods. In contrast, all 59, 56, and 2 macaques trapped in Kedah, Selangor, and W.P. Putrajaya, respectively tested negative both via BFMP and nested PCR (Table 1). All the 10% negative BFMP samples were also negative by PCR.

**Figure 3:**
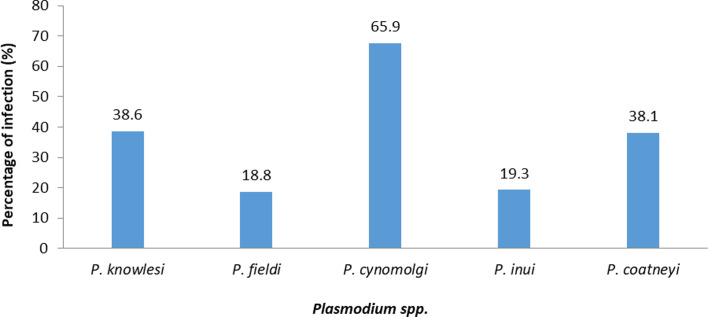
Macaque malaria as detected by Nested PCR. The most common *Plasmodium spp*. that was found in 176 selected Malaysian macaques was *P. cynomolgi,* followed by *P. knowlesi, P. coatneyi, P. fieldi,* and *P. inui.* Although *P. knowlesi* infection was not the most common in these macaques, it is by far the most fatal zoonotic malaria in humans.

All 202 samples that tested positive for the *Plasmodium* genus were subjected to species-specific identification. A total of 176 (87.13%) blood samples were positive for at least one of the 5 *Plasmodium spp*. in primates, including *P. knowlesi*, *P. fieldi*, *P. coatneyi*, *P. cynomolgi*, and *P. inui*. Despite being positive for both BFMP and genus PCR, 26 (12.87%) samples revealed no amplification for all five species. Among 176 samples tested positive for *Plasmodium spp.*, *P. cynomolgi* was the most predominant species [116 (65.9%)] followed by *P. knowlesi* [68 (38.6%)], *P. coatneyi* [67 (38.1%)], *P. inui* [34 (19.3%)] and *P. fieldi* [33 (18.8%)] (Fig. 3). Single and multiple infections were observed, whereby single infection was identified in 90 (51.1%) samples, followed by double, triple, and quadruple infections in 44 (25.0%), 30 (17.0%), and 10 (5.7%) samples, respectively. Interestingly, 2 (1.1%) samples revealed positive amplification for all 5 species. These findings are summarised in Table 2.

**Table 2:** Prevalence of simian malaria among macaque populations in Malaysia.

PCR result	Kelantan	Terengganu	Pahang	Sabah	W.P. Kuala Lumpur	Total
Single infection						
Pk	1	1	10	1		13
Pfd		1	4			5
Pcy			54	2	2	58
Pin		7				7
Pcty		1	6			7
Total	1	10	74	3	2	90
Double infection						
Pk + Pfd			2			2
Pk + Pcy			13	1		14
Pk + Pin			1			1
Pk + cty			3			3
Pfd + Pin		1	3			4
Pfd + Pcty			3			3
Pfd + Pcy			1			1
Pcy + Pin						0
Pcy + Pcty			14			14
Pin + Pcty		2				2
Total		3	40	1		44
Triple infection						
Pk + Pcy + Pcty			22			22
Pk + Pfd + Pin		1				1
Pk + Pin + Pcty		2				2
Pfd + Pin + Pcy			1			1
Pfd + Pin +Pcty		1	3			4
Total		4	26			30
Quadruplet infection						
Pk + Pfd + Pin + Pcty		1	5			6
Pk + Pfd + Pcy + Pin			2			2
Pfd + Pcy + Pin + Pcty			2			2
Total		1	9			10
Infection by all 5 species						
Pk + Pfd + Pcy + Pin + Pcty			2			2
Total			2			2
Total (%)	1 (0.6)	18 (10.2)	151 (85.8)	4 (2.3)	2 (1.1)	176 (100)

*Pk*, *P. knowlesi*; *Pfd*, *P. fieldi*; *Pcy*, *P. cynomolgi*; *Pin*, *P. inui*; *Pcty*, *P. coatneyi*.

### Phylogenetic analysis of *Plasmodium knowlesi*

The CSP genes were sequenced for samples with a single infection of *P. knowlesi*, including 10 samples from Pahang and one each from Kelantan, Terengganu, and Sabah. The reason that only *P. knowlesi* cases were sequenced is mainly because at this point in time, infections with *P. knowlesi* in humans were increasing and were more of a public health concern. Meanwhile, infection with other zoonotic simian malaria was very low or none. Phylogenetic analysis was limited to a single infection of *P. knowlesi*. The phylogenetic analysis was constructed to compared the genetic profile of CSP genes in human and macaques’ samples.

**Figure 4:**
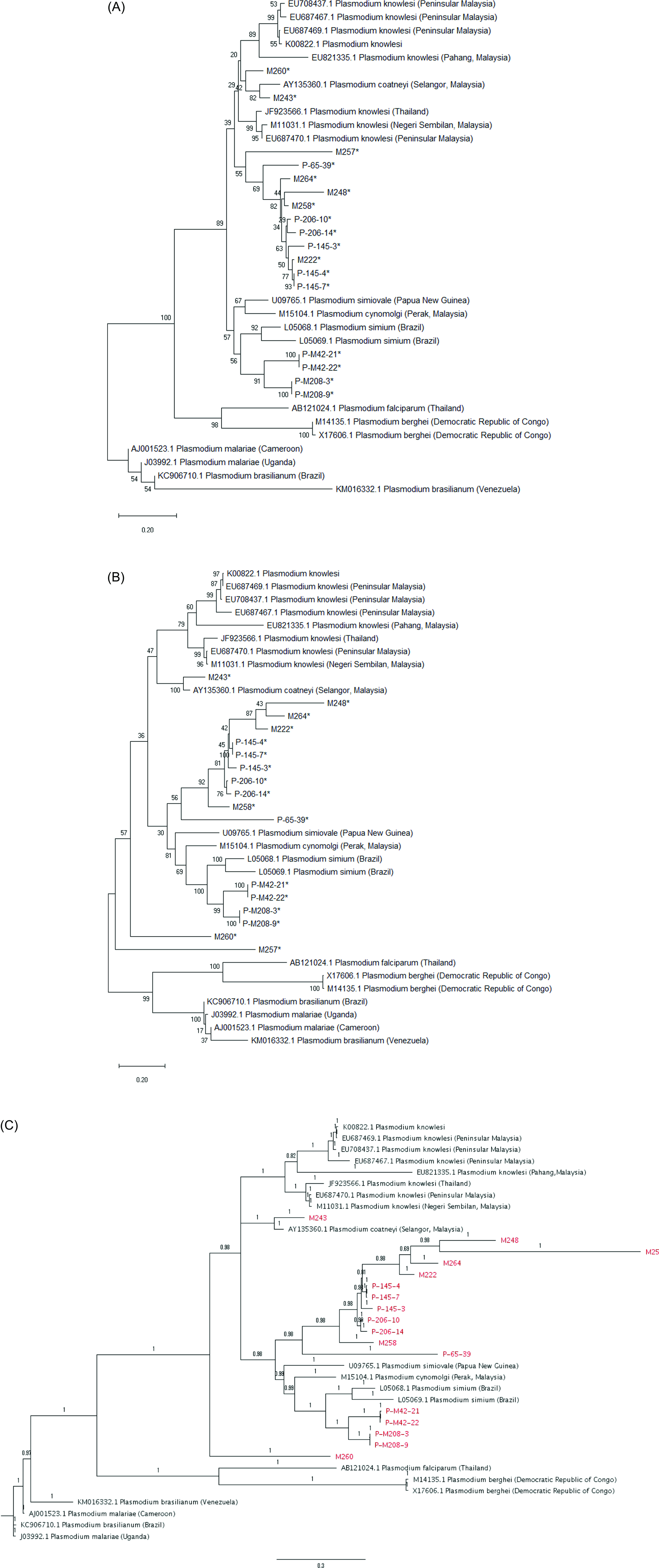
Phylogenetic tree based on CSP sequences of *Plasmodium* spp. All *P. knowlesi* CSP sequences obtained in this study are annotated as M257, M208 (P-M208-9-M13F, P-M208-3-M13F), M42 (P-M42-21-M13F, P-M42-22-M13F), M145 (P-145-7-M13F, P-145-4-M13F, P-145-3-M13F), M258, M206 (P-206-14-M13F, P-206-10-M13F), M65 (P-65-39-M13F), M260, M243, M222, M248, M264, and M234. The phylogenetic tree was constructed using the NJ method in MEGA7 (A), Bayesian (B) method using MrBayes software, and Maximum likelihood method (C) in MEGA-X. The percentages of replicate trees in which the associated isolates cluster together in the bootstrap test (1000 replicates) are shown next to the branches. The tree was drawn to scale, with branch length in the same unit as those of the evolutionary distances used to infer the phylogenetic tree. The evolutionary distances were computed using the Kimura 2-parameter and are in the units of the number of base substitutions per site.

The 13 CSP genes were amplified, cloned, and sequenced successfully. The PCR products ranged in size from 1050 to 1200 bp. The Neighbour Joining (NJ) method, Bayesian method, and Maximum-likelihood method inferred that malaria parasites isolated from these samples clustered with the reference *P. knowlesi* obtained from GenBank references in Table 3. Results of the phylogenetic analysis showed that the M257, M208 (P-M208-9-M13F, P-M208-3-M13F), M42 (P-M42-21-M13F, P-M42-22-M13F), M145 (P-145-7-M13F, P-145-4-M13F, P-145-3-M13F), M258, M206 (P-206-14-M13F, P-206-10-M13F), M65 (P-65-39-M) are related to or originated from the species found in Thailand and the East Coast of Peninsular Malaysia. The phylogenetic tree was created as a preventative measure to look for evidence of imported cases (Fig. 4).

**Table 3:** Reference sequence obtained from GenBank used in phylogenetic analysis.

Species	Country of origin	Accession number	Origin (man, mosquito, monkey, etc.)
*Plasmodium knowlesi*	Thailand	JF923566.1	Human
	Unknown	K00822.1	
	Peninsular Malaysia	EU687467.1	Human
		EU687469.1	Human
		EU687470.1	Human
		EU708437.1	Human
	Pahang, Malaysia	EU821335.1	Mosquito
	Negeri Sembilan, Malaysia	M11031.1	Monkey; *M. irus*
*Plasmodium coatneyi*	Selangor, Malaysia	AY135361.1	Mosquito; *A. hackeri*
*Plasmodium simium*	Brazil	L05068.1	Monkey; *Al. fusca, B. arachnoides*
		L05069.1	
*Plasmodium cynomolgi*	Perak, Malaysia	M15104.1	Monkey; *M. nemestrina*
*Plasmodium simiovale*	Papua New Guinea	U09765.1	Human
*Plasmodium berghei*	Democratic Republic of Congo	X17606.1	Unknown
		M14135.1	Unknown
*Plasmodium falciparum*	Thailand	AB121024.1	Unknown
*Plasmodium brasilianum*	Venezuela	KM016332.1	Human
	Brazil	KC906710.1	Monkey
*Plasmodium malariae*	Cameroon	AJ001523.1	Human
	Uganda	J03992.1	Chimpanzee

## Discussion

This study showed that simian malaria parasites are abundant in Malaysian macaques in certain states, with *P. cynomolgi* (67.6%) as the predominant species. However, the prevalence of *P. knowlesi* and *P. inui*, both of which have been shown to infect humans, is rather significant (38.6% and 19.3%, respectively). A previous study by Akter and co-workers identified *P. inui* as the most predominant species in 70 samples collected from Hulu Selangor, a district in the state of Selangor [1]. Akter’s study was concentrated in Hulu Selangor district, which is located in the north-eastern part of Selangor, bordering the state of Perak to the north, Pahang to the east, while our study was conducted in urban areas of Petaling, Gombak and Shah Alam districts indicated by the Wildlife Department as areas of monkey/human conflict, as this raises the risk of zoonotic transmission. It is well known that in Malaysia, Perak and Pahang states contributed to a great number of *P. knowlesi* cases in Peninsular Malaysia from 2019 to 2020 and infection could have been transmitted and spread to the neighboring districts and states (Source: Vector-Borne Disease Control, Ministry of Health, Malaysia). Similar results were also obtained from *M. fascicularis* samples in Sarawak [25] and Sabah [29]. In our survey, a large number of samples (419 samples) were collected from 6 states and two federal territories in Malaysia to generalize the infection distribution among the macaques. Although the original research plan was to include all states in Malaysia, logistic circumstances limited the collection of macaque blood samples to the aforementioned states. The compelling observation from these studies provides evidence that Malaysian *M. fascicularis* plays a significant role as a large reservoir of simian malaria parasites including those capable of zoonoses. This is in agreement with earlier twentieth-century parasitologists who suggested that macaques are the predominant simian host for malaria parasites [20].

Previous studies have documented an increasing number of malaria cases in Southeast Asia, with Malaysia having the highest number of *P. knowlesi* infections, which includes reports on the detection of *P. knowlesi* in humans especially in East Malaysia [36, 42, 46] and Peninsular Malaysia [50]. Cox-Singh and colleagues suggested that *P. knowlesi* is widely distributed in Malaysia, where the infection can potentially be life-threatening [13, 14]. However, most of the studies were focused mainly on infection by *P. knowlesi* in East Malaysia. Moreover, past studies focused more on the infection in humans with very limited critical assessments on the aspects involving macaques as the initial host. For example, Lee and co-investigators tackled the distribution of *P. knowlesi* infection in humans and *Plasmodium spp*. in macaques, but sample collection was limited to the state of Sarawak [25] which does not represent other major states in Malaysia.

Very recently, a survey was conducted to identify the prevalence of *Plasmodium spp.* in wild-caught macaques from three states of Peninsular Malaysia namely Pahang, Johor, and Perak [2]. The survey, however, extended the sampling to include pig-tailed macaques, *Macaca nemestrina,* whereby, consistent with our findings, *P. cynomolgi* was the most prevalent species [2]. The sampling sizes, as well as the unique geographical features of the states, may explain the difference in the prevalence of other *Plasmodium* species in the survey in comparison to ours [2]. Nonetheless, the results obtained from our survey serve as an addition, with a more evenly distributed sampling area involving the states in Peninsular Malaysia not included in the aforementioned survey [2]. The data from these two studies therefore provide the most extensive coverage of Peninsular Malaysia with samples collected in later years (from 2016 to 2019).

Due to an increasing number of human cases of *P. knowlesi* in Thailand, the Malaysian states neighboring Thailand, namely Kedah and Kelantan, were included as the sampling sites. The analysis of these samples could provide insights into the origin of infection according to geographical distribution. Interestingly, a similar study carried out among macaque populations in Southern Thailand showed the presence of only *P. inui* and *P. coatneyi* [30, 35]. In our study, priority was given to the analysis of CSP genes of *P. knowlesi* considering the life-threatening human disease it may cause. The phylogenetic analysis of *P. knowlesi* CSP genes indicated a high similarity to those of macaques, mosquitoes, and human samples isolated from the east coast region of Peninsular Malaysia and Thailand. Nevertheless, these results create a positive preliminary profile of the distribution of *P. knowlesi* in Malaysia and provide avenues for analysis of other simian malaria species in macaques with a high probability of future zoonotic transmission.

Although *P. cynomolgi* has been considered less important compared to *P. knowlesi*, the increasing evidence of its contribution to human health, as seen here, warrants further validation. For example, *P. cynomolgi* has long been known to be capable of infecting humans experimentally but in 2014, Ta and colleagues reported the first naturally acquired *P. cynomolgi* infection in humans [38]. However, more cases have been reported recently [18, 31, 37]. Molecular techniques may have helped in the identification. Otherwise, it may have been reported as *P. vivax*.

Meanwhile, the first naturally-acquired infection with *P. brasilianum* had been identified in one of the malaria-endemic regions in the Venezuelan Amazon, whereby 12 patients were found to be infected with *P. brasilianum* identical to those detected in macaques from the species *Alouatta seniculus*, as confirmed by sequencing of their CSP genes. Furthermore, an outbreak of *P. minimum* infection was reported in the Atlantic Forest in Rio de Janeiro following the conclusion that the cases of *P. vivax* for the past 50 years in the same areas were caused by *P. minimum* [4]. Malaria may in fact be a more difficult disease to curtail owing to the multiple distinct parasitic life cycle stages as well as its genetic complexity, which allows *Plasmodium* to adapt rapidly to drug pressure and challenges by the immune system [33].

An interesting finding in our study is that, in states where the number of *P. knowlesi* cases in humans was high, including Pahang, Kelantan, and Sabah [50], most of the macaques trapped were found to be hosts of *Plasmodium spp*. Correspondingly, in states with low cases of human *P. knowlesi*, such as Kedah and Terengganu [50], the detection of *Plasmodium spp.* in the respective trapped macaques was very low. While human cases of *P. knowlesi* infection were also detected in Selangor [50], none of the macaques trapped there were positive for any *Plasmodium spp.* This finding is also supported by a study by Braima and colleagues in 2013 [3]. They reported very comprehensively on an entomological survey during a study conducted to determine the risk of suburban transmission of malaria in Selangor state. In this study, they trapped most of the mosquitoes, mostly *Anopheles maculates*. However, none of the mosquitoes collected were shown to be positive wither with oocyst or sporozoites. The establishment of the relationship between the vectors (potential vectors) and the host still needs further investigation. Although the first naturally transmitted *P. cynomolgi* human infection was reported in Terengganu [38], macaques trapped in the same state were negative for *P. cynomolgi* but were infected with either single or mixed infection by *P. knowlesi*, *P. inui*, *P. coatneyi*, and *P. fieldi*. This may be due to the sampling areas. According to the report, the patient had been living and working in Hulu Terengganu, a district considered malaria-free [38]. However, long-tailed macaques and *Anopheles cracens* (the mosquito vector for *P. inui* and *P. cynomolgi*) were found to co-exist in the same area [38]. Out of 5 districts in Terengganu namely Besut, Kuala Terengganu, Hulu Terengganu, Dungun, and Kemaman, we only trapped and examined macaques in the first three districts. The human cases were from Hulu Terengganu district which bordered with Kelantan and Pahang states. Both states were known to be the highly endemic areas for *P. knowlesi* in Peninsular Malaysia.

The importance of identifying the distribution of simian malaria in macaques and the prevalence of zoonotic species lies in the risk of transmission to human populations, especially when there is clear evidence of vectors co-habiting in the same geographical areas. For instance, *P. knowlesi* infections were often misdiagnosed as *P. malaria* or *P. falciparum* [27]*. Plasmodium brasilianum* can also be identified as *P. malariae*, while *P. cynomolgi* and *P. simium* have a high resemblance to *P. vivax* [20]. The increase in the number of *P. knowlesi* cases and recent reports of infection by *P. cynomolgi*, *P. minimum,* and *P. brasilianum*, indicate that this may be due to conflicts between humans and macaques following rapid deforestation and land clearing for agriculture, infrastructure, real estate, and industry, as well as eco-tourism. Consequently, human populations have encroached into the vicinity of macaques’ natural habitat. On the other hand, improvements in molecular diagnostic methods and increased surveillance have increased detection of the infection, especially in endemic regions. Taken together, these preliminary results revealed a significant trend that could be helpful in learning and predicting the disease burden in the near future.

From this study we were able to provide insights on the prevalence of simian malaria in the long-tailed macaques collected, which may correlate to the increase of malaria zoonotic infection is in Malaysia, especially infection with *P. knowlesi* and now with *P. cynomolgi*. Technical reports by Vector-Borne Disease Centre (VBDC), Ministry of Health Malaysia in 2020 showed an increasing number of cases of *P. knowlesi* from 2008 to 2020 with mean cases of more than 3000. The highest number of cases was reported in 2018 with 4131 and slightly reduced in 2020 (2609 cases), which may be due to Movement Control Orders (MCOs) during the Covid-19 pandemic. The latest report on Malaria cases for the year 2000–2021 showed that total cases had increased from 2838 in 2020 to 3688 in 2021. In addition, more recent studies reveal that human infection with simian malaria was unquestionable and worrying, especially with *P. knowlesi* and *P. cynomolgi* [18, 28, 32, 49]. This supports the findings from local studies that have been carried out in the country that *Macaca fascicularis* is found to be the perfect host for this zoonotic *Plasmodium*, including for *P. knowlesi* and P*. cynomolgi*. This study has therefore strengthened the evidence in the past that implicated macaques as the potential source of increasing human infections. In addition, the information gathered has enhanced the evidence that previously implicated macaques are a possible source of rising human infections. If *P. knowlesi* is considered human malaria, failure to address these issues promptly may affect malaria elimination efforts Our findings could be exploited in any situation where predictions of outcomes are important. Furthermore, new research methodologies such as the use of a mathematical model, data mapping, and study on the sub-microscopic, could also be applied with caution to predict the upcoming disease burdens of simian malaria. This can be achieved by integrating all information on surveillance, the epidemiological link between disease-host-vector, genetic surveillance, and flexible modelling approach. Thus, it has become evident through our findings that certain precautions must be taken to be prepared for the upcoming disease burdens of simian malaria that, only in a matter of time, may have the potential to cause an outbreak. However, it remains to be further clarified whether our findings could be applied by policymakers to give stakeholders greater roles in planning necessary measures for malaria disease control and elimination. An integrated control strategy and multi-disciplinary agencies including experts from research institutes, universities, the wildlife department, the forestry department, and state government are required to discuss the strategy to manage this zoonotic disease, and analyze the situation at the human-animal-ecosystem interface. Without cooperation with all departments, these issues will be prolonged and new zoonotic malaria might be introduced to humans. Jessica Scott in her review in 2020 has suggested integrated control for zoonotic malaria where she highlighted some of the main issues that we need to take into consideration if we want to control or reduce zoonotic malaria [34]. The immediate measures that can be implemented are to disseminate knowledge and educate the population on the risk of infection with zoonotic malaria, and to travellers who take part in in eco-tourism, as well as to rural populations of the risk in zoonotic malaria-endemic areas: avoid close contact with macaques where cases of *P. knowlesi* are high and wear protective clothing, especially during peak mosquito biting hours.
